# The efficacy of progestins in managing pain associated with endometriosis, fibroids and pre-menstrual syndrome: a systematic review

**DOI:** 10.1007/s00404-025-07957-0

**Published:** 2025-03-11

**Authors:** Connor Luke Allen, Saikat Banerjee, Mahantesh Karoshi, Peter Humaidan, Farshad Tahmasebi

**Affiliations:** 1 Department of Medicine, Nursing and Health Sciences, Monash University, Melbourne, Australia; 2 Western Health, Melbourne, Australia; 3 CEES-U: Cambridge University Hospitals, Cambridge, UK; 4 Royal Free London NHS Foundation Trust, London, UK; 5 The Fertility Clinic, Skive Regional Hospital, Skive, Denmark; 6 Department of Clinical Medicine, Aarhus University, Aarhus, Denmark

**Keywords:** Analgesia, Progestins, Systematic review, Bone mineral density, Gynaecology

## Abstract

**Purpose:**

Alongside being contraceptives, progestins have been investigated as potential anti-inflammatory and analgesic therapies for use in painful gynaecological conditions. This review aims to synthesise evidence pertaining to the efficacy of progestins as analgesics for use in endometriosis, fibroids and pre-menstrual syndrome (PMS).

**Methods:**

We conducted a systematic review of the extant literature investigating the analgesic efficacy of progestins compared to any comparator interventions for individuals with the three specified gynaecological conditions. The search was carried out across the PubMed and CENTRAL databases on 7 April 2024 for randomised control trials (RCTs) published in the peer-reviewed literature from 2000 onwards. Data pertaining to analgesic efficacy, assessed by changes in pain indices/scores before and after treatment, were synthesised narratively. Data pertaining to adverse effect frequency and changes in bone mineral density (BMD) were also synthesised narratively. Risk of bias was assessed using the Cochrane risk of bias 2 tool.

**Results:**

The primary search identified 1220 potentially eligible RCTs of which 21 were ultimately included; 19 RCTs related to endometriosis, two related to fibroids and zero related to PMS. Quality assessment identified nine studies to be at a low risk of bias, nine studies with some concerns surrounding bias and three studies to be at a high risk of bias. The included studies represented a total of 2745 participants of whom 1317 were treated with a progestin and 1428 received a comparator intervention. In 18 of the 19 studies concerning endometriosis, progestins produced a statistically significant reduction in pain, further, in five instances progestins were more efficacious in reducing pain than comparator interventions. In both studies on fibroids, progestins produced significant reductions in pain, however, statistically significant differences compared to comparator interventions were not demonstrated. The most frequently cited adverse effect of progestins was spotting/irregular bleeding whilst those receiving comparator interventions most often reported hot flushes; cited in 12 and seven studies respectively. Five studies assessed the impact of progestins and comparators on BMD. Three studies found progestins significantly reduced BMD, however, in these instances reductions were significantly lower than those produced by comparator interventions and in two studies were not statistically significant after 12 months of follow-up.

**Conclusion:**

Our review demonstrates the potential scope for the use of progestins as analgesics in the management of pain associated with endometriosis. Further research will need to be conducted to identify their efficacy in the management of pain associated with fibroids and PMS.

**Supplementary Information:**

The online version contains supplementary material available at 10.1007/s00404-025-07957-0.

## Introduction

Progestins are synthetic analogues of the hormone progesterone that act by binding to and agonising progesterone receptors in reproductive tissues [1]. Agonism of progesterone receptors suppresses hypothalamic production of gonadotrophin-releasing hormone (GnRH), subsequently downregulating secretion of luteinising hormone (LH) and follicle-stimulating hormone (FSH) from the pituitary gland [2]. Reduced levels of LH and FSH interfere with the menstrual cycle by inducing ovulation suppression, which ultimately results in amenorrhoea [2]. Progestins’ amenorrheic properties and suitability as contraceptives are further enhanced through mechanisms such as thickening of the cervical mucus and alteration of the endometrial lining [3, 4].

In addition to their contraceptive effects, progestins have been demonstrated to possess anti-inflammatory properties through a variety of proposed mechanisms. Studies have elucidated the ability of progestins to inhibit inflammatory pathways via suppression of cyclooxygenase-2 (COX-2), an enzyme central to the synthesis of prostaglandins, which are involved in the inflammatory response [5]. Downregulation of COX-2 and subsequent reductions in pro-inflammatory prostaglandins ultimately mitigates inflammation [5]. Concurrently, progestins have been demonstrated to inhibit the release of pro-inflammatory cytokines and chemokines from stromal tissue, including interleukin (IL)-6, IL-8 and monocyte chemotactic protein (MCP)-1, thereby reducing inflammation [6]. Progestins have also been shown to modulate nuclear factor-kappa B, a transcription factor that influences the expression of multiple pro-inflammatory genes that drive the systemic inflammatory response [7]. These anti-inflammatory properties suggest progestins might be favourable for use as both contraceptives and analgesic therapies for inflammation-related pain associated with a range of gynaecological conditions.

One of the most prevalent and painful gynaecological diseases for women and people with uteri is endometriosis, a chronic disease characterised by endometrial-like tissue existing outside of the uterus [8]. This tissue is typically situated in other pelvic anatomy including the ovaries and pelvic peritoneum but also potentially in extra-pelvic sites throughout the body [9]. Endometriosis causes a significant global burden of disease, The World Health Organisation (WHO) estimates that 10% of women of reproductive age worldwide (roughly 190 million) are affected, many of whom experience symptoms including dysparaeunia, dysmenorrhoea and infertility [10, 11]. Currently, medical therapy consisting of progestin-only and combined oestrogen-progestin (OCP) formulations is thought to be successful in treating up to two-thirds of pain associated with endometriosis [12]. This has been attributed to the ability of these therapies to induce atrophy of endometrial tissue and to produce synergistic anti-inflammatory and proapoptotic effects [12]. However, safety concerns surrounding sustained use of OCPs, and their demonstrated thrombosis risk are longstanding. For example, a 2014 systematic review of 26 studies identified that the use of OCPs was associated with a 3.5 times higher risk for the development of venous thrombosis compared to no therapy [13]. Comparatively, progestin-only therapies do not appear to increase thrombosis risk, suggesting their potential utility in patients where risks associated with OCP are unacceptable [14]. Alongside the thrombosis risk, the efficacy of OCPs as a treatment for endometriosis-associated pain also remains unclear [15].

Uterine fibroids or leiomyoma are benign smooth muscle neoplasms situated in the uteri of women and people of reproductive age [16]. Fibroids are capable of precipitating significant pain in the pelvis and other anatomy, such as bowel and bladder, which frequently coexists with symptoms including menorrhagia, irregular menstruation and abdominal cramping [17–19]. Fibroids continue to represent a significant gynaecological problem, with a 2018 systematic review examining data from 60 studies identified that fibroids occur in approximately 70% of women, with disproportionate predominance in Black women [20]. While there is evidence that progesterone plays a role in fibroid growth, the role that progestin therapies have to play in managing pain associated with fibroids remains unclear [21, 22].

Premenstrual syndrome (PMS) is characterised by physical discomfort and mood symptoms in the early, mid or late luteal phase immediately preceding menstruation [23]. Globally, PMS is thought to affect between 5 and 8% of women [23]. Whilst several mechanisms have been proposed, the pathophysiology of PMS remains undetermined [24]. Symptoms associated with PMS are closely related to ovarian progesterone production, indicating a likely hormonal element [25]. A 2012 Cochrane review of two studies suggested that there was some evidence for progesterone as an effective agent for the management of PMS symptoms, however, stated that the methodology and management of outcome data in these studies were flawed [26].

Whilst systematic reviews examining the analgesic efficacy of progestins in managing endometriosis have been published, these relied heavily on data from observational studies [27] and/or a limited number of studies (four RCTs) [28]. Furthermore, no recent systematic reviews have sought to compare this analgesic efficacy across multiple gynaecological conditions. This review seeks to synthesise evidence for the use of progestins versus comparator interventions for the management of pain in individuals with endometriosis, fibroids and PMS using data from RCTs published since 2000. By synthesising these data, this review aims to clarify the therapeutic potential of progestins as analgesics for these conditions and to address conflicting data and gaps in the literature.

## Methods

We conducted a systematic review of the literature to identify RCTs that assessed the efficacy of progestins versus comparator interventions for pain management in individuals with endometriosis, fibroids and PMS. This review was conducted in accordance with the PRISMA 2020 Statement (Online Resource 1) and was registered on PROSPERO with the ID: CRD42024533148 on 7 April 2024 [29].

### Eligibility criteria

RCTs, both parallel and cross-over, published since 2000 were eligible to be included in this review. The restriction to include only studies published since 2000 was instated to ensure that included studies were representative of contemporary practices and therapies. RCTs were not strictly required to be multi-site or placebo-controlled to be eligible for inclusion. No language or age restrictions were imposed. All synthetic progesterone/progestin therapies currently approved for use in humans were eligible for inclusion.

The following types of studies were excluded:

- Studies that only evaluated progestins as components of combination therapies (e.g., oestrogen/progesterone).
- Pilot studies, patient preference trials and extension studies.
- Conference abstracts and posters.
- Observational studies e.g., cohort and case–control studies.
- Review articles were ineligible, however, relevant systematic reviews were screened for additional relevant studies.
- Studies that did not clearly delineate pain improvement as a distinct clinical outcome or as a component of a composite outcome measure.
- Trials that examined the role of progestin therapy in pain improvement outside of the scope of the three conditions of focus.

### Search strategy and screening

**Fig. 1:**
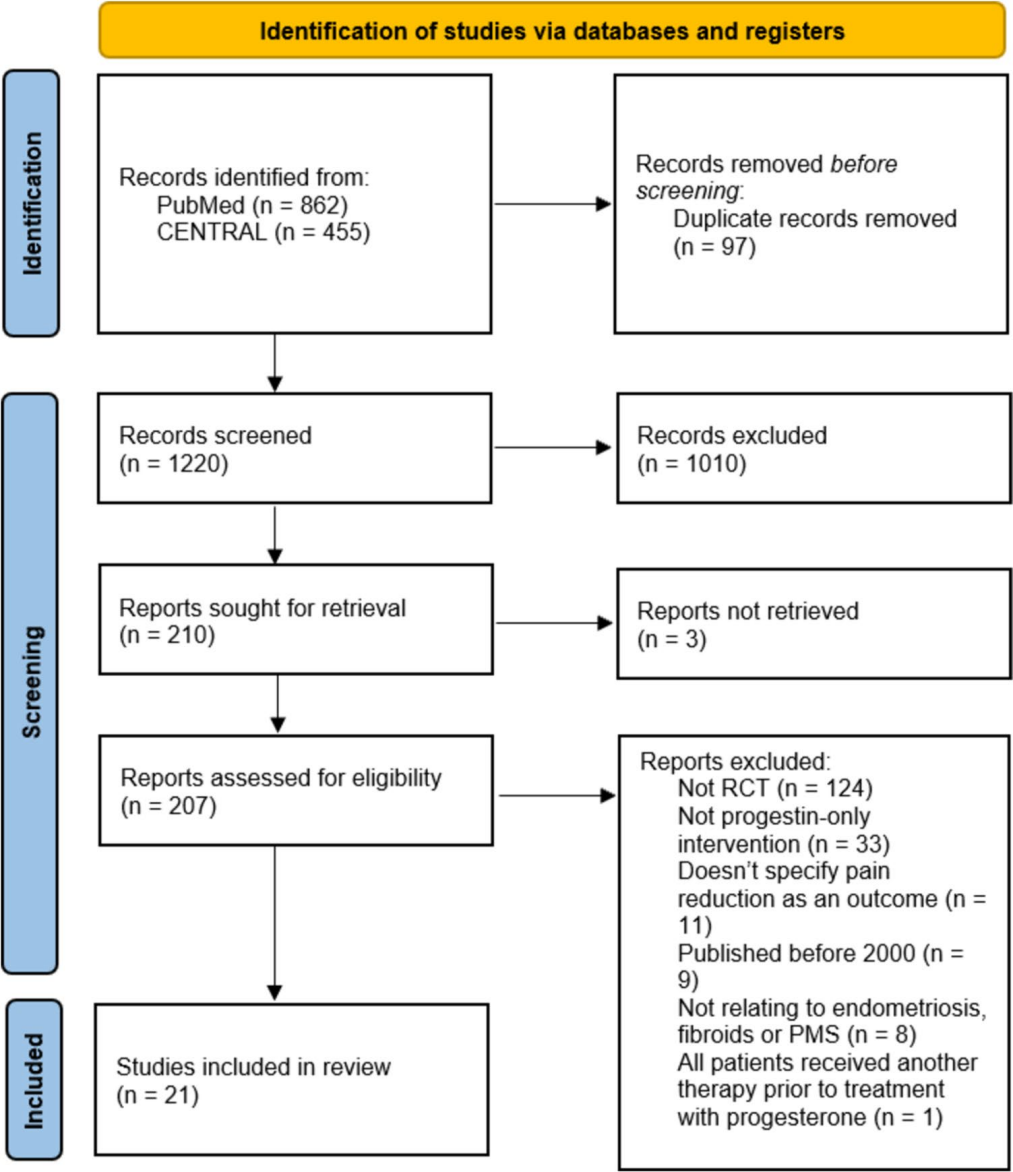
PRISMA flowchart of the study selection process. Study flow diagram sourced from: Page MJ, McKenzie JE, Bossuyt PM, Boutron I, Hoffmann TC, Mulrow CD, et al. The PRISMA 2020 statement: an updated guideline for reporting systematic reviews. BMJ 2021;372:n71. https://doi.org/10.1136/bmj.n71.

A structured search of the literature was conducted with the intention of identifying RCTs that encompassed our three broad domains of inquiry: pain reduction, the three gynaecological conditions of focus (endometriosis, fibroids and PMS) and progestin therapies. The search strategies executed across the PubMed and CENTRAL databases on 7 April 2024 are attached as Online Resource 2. The results of this search were subsequently imported to Endnote and uploaded to the Covidence platform (https://app.covidence.org/) where retrieved studies were automatically de-duplicated. Retrieved studies were then screened by two authors against eligibility criteria first by title/abstract and subsequently by full text. The PRISMA flowchart outlining the studies included at each stage of the screening and selection process is included in Fig. 1.

### Data extraction

Data were extracted by one author and verified by another with disagreements resolved via discussion. Characteristics of each of the included studies were extracted including author(s), year of publication, country/countries conducted in, participant characteristics, number of participants assigned to each arm and their mean ages (with standard deviation). Pertinent clinical information was collected including details of therapies evaluated e.g., pharmacological agent(s), dose(s), frequency, route of administration and duration of treatment. The clinical outcomes and effect measures of interest, in particular composite pain scores and author conclusions, were tabulated. Statistical significance, as referred to in this review, is as stated by each of the included studies. Meta-analysis was not possible due to the inclusion of multiple conditions and marked variability in study protocols, drugs, dosages and routes of administration, thus, results were analysed narratively.

### Quality assessment

Quality assessment was performed by two authors independently with disagreements resolved via discussion. Quality of the included trials was assessed using the Cochrane risk of bias (RoB) 2 tool wherein the following data were evaluated: method of randomisation, allocation concealment, blinding, completeness of data and selective reporting [30]. Studies were scored either ‘low’, ‘some concerns’, or ‘high’ in each of these domains to comprise an overall rating. To ensure maximum relevant data were identified for inclusion, studies were not excluded on the basis of their deemed quality.

## Results

### Summary of included studies

We identified 1220 potentially eligible RCTs at title/abstract screening of which 21 [31–51] were eligible for inclusion in this review. This selection process is displayed visually in Fig. 1. 19 studies [31–49] examined the analgesic efficacy of progestins for endometriosis-associated pain whilst a further two studies [50, 51] did so for fibroid-related pain. We did not identify any studies, published since 2000, examining the analgesic efficacy of progestins for PMS-associated pain. Details of studies that appeared to meet inclusion criteria that were ultimately excluded are available in Online Resource 3. The included studies represented a total of 2745 participants of whom 1317 were treated with a progestin and 1428 who received a comparator intervention. Participants ranged in mean age from 18 to 42.7 years. Studies were geographically diverse and predominantly conducted in high-income and upper-middle-income countries; only one study [38] was conducted in a lower-middle-income country, none originated from low-income countries. Detailed characteristics of included studies are outlined in Table 1.

**Table 1:** Characteristics of included RCTs

Author (year)	Country	Participant characteristics	Relevant sample size	Treatment duration	Progestin (*n*)	Comparator (*n*)	Comparator (*n*)	Mean age (years)	Outcome measures	Risk of bias
Bayoglu Tekin et al., (2011) [31]	Turkey	Women with severe endometriosis indicated by a score of at least 40 on the revised American Society for Reproductive Medicine score (rASRM)	40	12 months	Levonorgestrel intrauterine system (LNG-IUS) (*n* = 20)	Goserelin acetate (monthly; IM) (*n* = 20)	–	36.5 ± 4.5 (Dienogest) 38.7 ± 4.8 (Goserelin) *p* > 0.05	Visual Analogue Scale (VAS) Total Endometriosis Severity Profile (TESP)	Low
Carr et al., (2014) [32]	United States	Women with laparoscopically diagnosed endometriosis with a Composite Pelvic Signs and Symptoms Score (CPSSS) of at least six with a dysmenorrhoea score and a nonmenstrual pelvic pain score of at least two	251	24 weeks	Depot medroxyprogesterone acetate (DMPA) (104 mg/0.65 ml/12 weekly; subcutaneously (SC)) (*n* = 83)	Elagolix (150 mg/daily; orally) (*n* = 84)	Elagolix (75 mg/twice daily; orally) (*n* = 84)	31.6 ± 0.4 (DMPA) 32.4 ± 0.8 (Elagolix 150 mg) 31.4 ± 0.7 (Elagolix 75 mg) No p-value, ‘similar’	Bone mineral density (BMD) CPSSS (modified from the Biberoglu and Behrman (B&B) Scale) VAS Endometriosis Health Profile-5 (EHP-5) Uterine bleeding and hot flash assessments	Low
Caruso et al., (2022) [33]	Italy	Women with endometriosis-associated chronic pelvic pain, dysmenorrhoea and dysparaeunia	197	12 months	Dienogest (2 mg/daily; orally) (*n* = 98)	OCP (17b-estradiol 1.5 mg and nomegestrol acetate 2.5 mg/daily; orally) (*n* = 99)	–	18 – 39 (Dienogest) 18 – 38 (OCP) *p* = 1.0	VAS Short-Form-36 (SF-36) Female Sexual Function Index (FSFI) Female Sexual Distress Scale (FSDS)	Some concerns
Carvalho et al., (2018) [34]	Brazil	Women with surgically diagnosed endometriosis stages I-IV (rASRM) or women with a diagnosis of deep endometriosis on imaging and complaints of non-cyclic chronic pelvic pain and dysmenorrhea or both for more than six months	103	6 months	LNG-IUS (*n* = 51)	ENG implant (*n* = 52)	–	34.7 ± 0.925 (LNG-IUS) 33.4 ± 0.892 (ENG implant) *p* = 0.286	VAS Endometriosis Health Profile-30 (EHP-30)	Some concerns
Ceccaroni et al., (2021) [35]	Italy	Women who had undergone laparoscopic eradication of deep infiltrating endometriosis stages III and IV (rASRM)	146	6 months	Dienogest (2 mg/daily; orally) (*n* = 65)	GnRH agonist (Triptorelin or Leuprorelin) (3.75 mg/4 weekly; orally) (*n* = 81)	–	34 ± 5.5 (Dienogest) 34 ± 5.5 (GnRH agonist) No *p*-value	VAS Treatment tolerability Imaging relapse rate Pregnancy rate	Some concerns
Cheewadhanaraks et al., (2012) [36]	Thailand	Women who had undergone conservative surgery for symptomatic endometriosis	84	24 weeks	DMPA (150 mg/12 weekly; IM) (*n* = 42)	OCP (ethinyl estradiol 0.03 mg and gestodene 0.075 mg/daily; orally) (*n* = 42)	–	30.5 ± 5.4 (OCP) 31.9 ± 5.5 (DMPA) No *p*-value, ‘similar’	VAS Verbal Rating Scale (VRS) (modified from B&B Scale)	Some concerns
Crosignani et al., (2006) [37]	Multiple	Women with laparoscopically diagnosed endometriosis and persistent pain symptoms	299	24 weeks	DMPA (104 mg/0.65 ml/3 monthly; SC) (*n* = 153)	Leuprolide (3.75 mg/monthly; orally or 11.25 mg/3 monthly; orally) (*n* = 146)	–	31.8 ± 6.7 (DMPA) 30.9 ± 6.1 (Leuprolide) No *p*-value, ‘similar’	B&B Scale EHP-30 SF-36 Patient Satisfaction Questionnaire BMD Kupperman Index	Low
El Taha et al., (2021) [38]	Lebanon	Women with histologically confirmed endometriosis stages I-IV (rASRM) and/or endometriosis diagnosed on imaging plus complaints of dysmenorrhoea and/or non-cyclic chronic pelvic pain for more than six months	70	24 weeks	Dienogest (2 mg/daily; orally) (*n* = 35)	OCP (ethinyl estradiol 0.03 mg and drospirenone 3 mg/daily; orally) (*n* = 35)	–	28.3 ± 6.5 (Dienogest) 29.8 ± 6.5 (OCP) *p* = 0.343	VAS B&B Scale EHP-30	Low
Ferreira et al., (2010) [39]	Brazil	Women with laparoscopically and histologically diagnosed endometriosis with chronic pelvic pain	44	6 months	LNG-IUS (*n* = 22)	Leuprolide (3.75 mg/monthly; IM) (*n* = 22)	–	28.8 ± 4.9 (LNG-IUS) 31.4 ± 5.8 (Leuprolide) *p* = 0.14	VAS	Some concerns
Harada et al., (2009) [40]	Japan	Women with endometriosis diagnosed by laparotomy, laparoscopy or imaging analysis in the presence of symptoms	271	24 weeks	Dienogest (2 mg/daily; orally) (*n* = 137)	Buserelin acetate (900μg/daily; intranasally) (*n* = 134)	–	33.5 ± 6.9 (Dienogest) 33.8 ± 6.2 (Buserelin acetate) *p* > 0.05	VAS SF-36 Subjective symptoms during non-menstruation BMD	Low
Lang et al., (2018) [41]	China	Women with a diagnosis of endometriosis confirmed by laparoscopy or laparotomy within ten years before study entry and a VAS score of at least 30 for EAPP over the last four weeks	255	24 weeks	Dienogest (2 mg/daily; orally) (*n* = 126)	Placebo (daily; orally) (*n* = 129)	–	35.5 ± 5.02 (Dienogest) 35.1 ± 5.05 (Placebo) No *p*-value	VAS (EAPP) B&B Scale Bleeding patterns Cervical smear Transvaginal/abdominal ultrasound BMD SF-36	Low
Margatho et al., (2020) [42]	Brazil	Women with surgically or histologically confirmed endometriosis stages I-IV (rASRM) or a diagnosis of deep endometriosis on imaging with chronic pelvic pain and/or dysmenorrhoea for more than six months and a VAS score of at least four	103	24 months	LNG-IUS (*n* = 51)	Etonogestrel implant (ENG) (*n* = 52)	–	34.7 ± 0.93 (LNG-IUS) 33.4 ± 0.89 (ENG) *p* = 0.286	VAS	Low
Razzi et al., (2007) [43]	Italy	Women with mild, histologically confirmed endometriosis stages I-II (rASRM) following laparoscopic ovarian cystectomy	40	6 months	Desogestrel (75 μg/daily; orally) (*n* = 20)	OCP (Ethinyl estradiol 20μg and desogestrel 150μg/daily; orally) (*n* = 20)	–	25 – 35 (Desogestrel) 23 – 34 (OCP) No *p*-value, ‘similar’	VAS	Some concerns
Regidor et al., (2001) [44]	Germany	Women with histologically confirmed endometriosis stages I-IV (rASRM)	48	6 months	Lynestrenol (5 mg/twice daily; orally) (*n* = 22)	Leuprorelin acetate depot (3.75 mg/monthly; SC) (*n* = 26)	–	30.9 ± 5.7 (Lynestrenol) 32.4 ± 6.5 (Leuprorelin) No *p*-value	rASRM scores B&B Scale	Some concerns
Schlaff et al., (2006) [45]	Canada & United States	Women with laparoscopically confirmed endometriosis with persistent pain symptoms	274	6 months	DMPA (104 mg/0.65 mL/3 monthly; SC) (*n* = 136)	Leuprolide (11.25 mg/3 monthly; IM) (*n* = 138)	–	29.2 ± 6.3 (DMPA) 32.1 ± 6.6 (Leuprolide) *p* < 0.001	B&B Scale SF-36 BMD	Low
Strowitzki et al., (2010) [46]	Germany, Italy & Ukraine	Women with laparoscopically confirmed endometriosis stages I-IV (rASRM) with an EAPP score of at least 30	198	12 weeks	Dienogest (2 mg/daily; orally) (*n* = 102)	Placebo (daily; orally) (*n* = 96)	–	31.5 ± 6.7 (Dienogest) 31.4 ± 6.0 (Placebo) No *p*-value	VAS (EAPP) B&B Scale Change in intake of supportive analgesic medication SF-36	High
Tanmahasamut et al., (2012) [47]	Thailand	Women with endometriosis and moderate‐to‐severe dysmenorrhea who have undergone laparoscopic surgery	55	12 months	LNG-IUS (*n* = 28)	Expectant management (*n* = 27)	–	33.4 ± 6.7 (LNG-IUS) 32 ± 8.6 (Expectant management) *p* = 0.492	VAS SF-36	Some concerns
Tanmahasamut et al., (2017) [48]	Thailand	Women with laparoscopically treated endometriosis with moderate-to-severe dysmenorrhea and/or chronic pelvic pain for more than six months	40	24 weeks	Desogestrel (0.075 mg/daily; orally) (*n* = 20)	Placebo (daily; orally) (*n* = 20)	–	29.1 ± 4.9 (Desogestrel) 32.7 ± 6.7 (Placebo) *p* = 0.059	VAS Number of rescue medications	Low
Vercellini et al., (2005) [49]	Italy	Women who had undergone laparoscopy or laparotomy for stage I-IV (rASRM) endometriosis in the past 12 months and in whom rectovaginal lesions were not removed	90	12 months	Norethindrone acetate (2.5 mg/daily; orally) (*n* = 45)	OCP (Ethinyl estradiol 0.01 mg and cyproterone acetate 3 mg/daily; orally) (*n* = 45)	–	Not specified No *p*-value, ‘similar’	VAS VRS Mean volume of rectovaginal plaques	Some concerns
Fibroids										
Verspyck et al., (2000) [50]	France	Women with symptomatic uterine myomas with ultrasonographic evidence of one or more myomas of at least five cm diameter or a submucous myoma	56	16 weeks	Lynestrenol (5 mg/twice daily; orally) (*n* = 23)	Leuprorelin (3.75 mg/monthly; IM) (*n* = 33)	–	40.17 ± 1.69 (Lynestrenol) 42.24 ± 1.27 (Leuprorelin) *p* = 0.37	Myoma size VAS	High
Whitaker et al., (2023) [51]	United Kingdom	Women with heavy menstrual bleeding including women with fibroids	81	12 months	LNG-IUS (*n* = 38)	Ulipristal acetate (UPA) (5 mg/daily for 3, 12-week cycles separated by 4-week treatment free intervals; orally) (*n* = 43)	–	42.4 ± 6.9 (LNG-IUS) 42.7 ± 7 (UPA) No *p*-value, ‘similar’	Uterine Fibroid Symptom and Quality of Life (UFS-QOL) Menorrhagia Multi-Attribute Scale Questionnaire (MMAS) Bleeding diary scores	High

Quality assessment determined using Cochrane RoB 2 [30] delineated nine studies [31, 32, 37, 38, 40–42, 45, 48] to be at a low risk of bias, nine studies [33–36, 39, 43, 44, 47, 49] with some concerns surrounding bias and three studies [46, 50, 51] at a high risk of bias. The most common source of bias in the included studies was due to missing outcome data. Results of the quality assessment are outlined in Table 1 and visible displayed graphically in Online Resource 4.

### Interventions compared

A range of progestins, routes of administration, doses and durations of treatment were represented across the included studies. The progestins evaluated by each study were dienogest (*n* = 6), levonorgestrel-releasing intrauterine system (LNG-IUS) (*n* = 6), depot medroxyprogesterone acetate (DMPA) (*n* = 4), desogestrel (*n* = 2), lynestrenol (*n* = 2) and norethindrone acetate (*n* = 1). The most common comparators were GnRH agonists (*n* = 8), these included leuprolide (*n* = 3), leuprorelin (*n* = 2), leuprorelin/triptorelin (*n* = 1), goserelin (*n* = 1), buserelin (*n* = 1). Other comparators included combined oral contraceptives (*n* = 5), placebo (*n* = 3), etonogestrel implant (*n* = 2), elagolix (*n* = 2), UPA (*n* = 1), and expectant management (*n* = 1). The most frequent progestin/comparator combination was dienogest versus a GnRH agonist (*n* = 3).

### Pain scales and indices used

The efficacy of interventions for reducing pain was measured in a variety of ways and differed between studies related to endometriosis and fibroids. The most common tool by which the efficacy of pain reduction was measured for endometriosis was by utilising changes in Visual Analogue Scale (VAS) scores (millimetres [mm]) pre- and post-treatment. A total of 17 endometriosis-related studies and one fibroid-related study used the VAS in isolation and/or in conjunction with another tool to evaluate changes in pain before and after treatment. Other tools used to measure changes in pain included the Biberoglu and Behrman Scale (*n* = 6) and the Verbal Rating Scale (VRS) (*n* = 2), all other pain measurement tools appeared only once. Of the two studies pertaining to fibroids, one measured pain reduction with the VAS and the other with the Uterine Fibroid Symptom and Quality of Life Questionnaire (UFS-QOL). Safety of agents was often described in relation to changes in bone mineral density (BMD), ultimately five studies [32, 37, 40, 41, 45] analysed this. Adverse effects were represented and collated in various ways across studies, full details are outlined in Table 3.

### Summary of efficacy

Only one study [31] found that progestins did not produce statistically significant improvements in pain scores after completion of treatment. All other studies demonstrated that progestins significantly reduced pain associated with endometriosis or fibroids. Full details of efficacy are outlined in Table 2.

**Table 2:** Pain reduction outcomes

Author (year)	Progestin (*n*)	Comparator (*n*)	Comparator (*n*)	Pain outcome measure	Pain improvement outcomes	Author conclusions
Endometriosis						
Bayoglu Tekin et al., (2011) [31]	Levonorgestrel intrauterine system (LNG-IUS) (*n* = 20)	Goserelin acetate (monthly; IM) (*n* = 20)	–	Visual analogue scale (VAS) Total endometriosis severity profile (TESP)	VAS (chronic pelvic pain): - LNG-IUS group did not show a significant difference at 12 months follow up (*p* > 0.05) - Goserelin acetate significantly reduced VAS at 12 months follow up (*p* = 0.048) - No interarm comparison TESP (dysmenorrhoea and dysparaeunia): - LNG-IUS: significant reduction in TESP scores at months one, three and six but elevated to above pre-treatment levels at 12 months follow up (*p* > 0.05) - Goserelin acetate: significant reduction in TESP scores at 12 months follow up (*p* < 0.001)	At 12 months only goserelin acetate, not LNG-IUS, significantly reduced TESP and VAS scores
Carr et al., (2014) [32]	Depot medroxyprogesterone acetate (DMPA) (104 g/0.65 ml/12 weekly/SC) (*n* = 83)	Elagolix (150 mg/daily; orally) (*n* = 84)	Elagolix (75 mg/daily; twice daily) (*n* = 84)	CPSSS (modified from the B&B Scale) VAS	CPSS (dysmenorrhoea and non-menstrual pelvic pain) mean change in scores from baseline to 24 weeks: - Elagolix 150 mg/daily: − 5.50 ± 0.34 (*p* < 0.05) - Elagolix 75 mg/bd: − 5.20 ± 0.32 (*p* < 0.05) - DMPA: − 5.30 ± 0.36 (*p* < 0.05) VAS (pelvic pain): - All arms showed improvement in VAS scores for pelvic pain from baseline. Elagolix 75 mg/bd showed the most pronounced effects	Elagolix 150 mg/daily was statistically noninferior to DMPA in treating the dysmenorrhea and non-menstrual pelvic pain components of the CPSSS at 24 weeks
Caruso et al., (2022) [33]	Dienogest (DNG) (2 mg/daily; orally) (*n* = 98)	Combined oral contraceptive (OCP) (17b-estradiol 1.5 mg and nomegestrol acetate 2.5 mg/daily; orally) (*n* = 99)	–	VAS	VAS (chronic pelvic pain, dysmenorrhoea and dysparaeunia): - Both arms showed improvement of the VAS score between baseline and at 12 months (*p* < 0.001) - Comparison between arms showed no significant difference at baseline (*p* = 0.08), at 3 months (*p* = 0.06) or at 12 months (*p* = 0.06) - Only at 6 months did DNG show a statistically significant improvement in symptoms compared to the OCP group (*p* = 0.01)	At 12 months both groups showed improvement in VAS scores; differences between the two groups were not significant
Carvalho et al., (2018) [34]	LNG-IUS (*n* = 51)	ENG implant (*n* = 52)	–	VAS	VAS (non-cyclic pelvic pain): - Both interventions significantly reduced non-cyclic pelvic pain at 180 days - ENG: baseline 7.6 ± 1.7 (95% CI 7.1, 8.0) to 2.0 ± 2.4 (95% CI 1.2, 2.7), mean difference: 5.6 ± 1.7 (95% CI − 6.4, − 4.7; *p* < 0.0001) - LNG-IUS: baseline 7.4 ± 1.7 (95% CI 6.9, 7.9) to 1.9 ± 1.7 (95% CI 1.3, 2.4), mean difference: 5.5 ± 1.6 (95% CI − 6.2, − 4.4; *p* < 0.0001) VAS (dysmenorrhoea): - Both interventions significantly reduced dysmenorrhoea at 180 days - ENG: baseline 7.5 ± 1.7 (95% CI 6.9, 8.1) to 2.2 ± 3.2 (95% CI 1.1, 3.2), mean difference: 5.3 ± 1.3 (95% CI − 6.6, − 4.3; p < .0001) - LNG-IUS: baseline 7.3 ± 1.7 (95% CI 6.9, 7.9) to 1.9 ± 2.2 (95% CI 1.2, 2.7), mean difference: 5.4 ± 1.3 (95% CI − 6.3, − 4.3; *p* < .0001) There was no significant difference between arms for improving non-cyclic pelvic pain and dysmenorrhoea 0.01 ± 0.72 (95% CI − 1.10, 1.14; no p-value)	Both interventions improved the mean VAS scores with no significant difference between groups
Ceccaroni et al., (2021) [35]	Dienogest (2 mg/daily; orally) (*n* = 65)	GnRH agonist (Triptorelin or Leuprorelin) (3.75 mg/4 weekly; orally) (*n* = 81)	–	VAS	VAS (overall): - Both arms significantly reduced pain between baseline and at six months (*p* < 0.001) - Both arms significantly reduced pain between baseline and at 30 ± 6 months (*p* < 0.001) VAS (dysmenorrhoea): - DNG: baseline 70.9 to 4.00 at six months to 13.2 at 30 months (*p* < 0.001) - GnRH agonist: baseline 75.8 to 0.60 at six months to 4.50 at 30 months (*p* < 0.001) - No statistical difference between groups at six or 30 months VAS (dysparaeunia): - DNG: baseline 48.4 to 3.00 at six months to 7.30 at 30 months (*p* < 0.001) - GnRH agonist: baseline 45.9 to 1.40 at six months to 2.80 at 30 months (*p* < 0.001) - No statistical difference between groups at six or 30 months VAS (dyschezia): - DNG: baseline 42.6 to 8.30 at six months to 8.10 at 30 months (*p* < 0.001) - GnRH agonist: baseline 48.7 to 3.50 at six months to 4.80 at 30 months (*p* < 0.001) - No statistical difference between groups at six and 30 months VAS (chronic pelvic pain): - DNG: baseline 41.9 to 5.00 at six months to 9.20 at 30 months (*p* < 0.001) - GnRH agonist: baseline 47.7 to 2.20 at six months to 3.80 at 30 months (*p* < 0.001) - No statistical difference between groups at six and 30 months VAS (low back pain): - DNG: baseline 14.9 to 2.40 at six months to 3.50 at 30 months (*p* < 0.01) - GnRH agonist: baseline 8.80 to 0.50 at six months to 0.60 at 30 months (*p* < 0.01) - No statistical difference between groups at six and 30 months VAS (dysuria): - DNG: baseline 15.5 to 0.10 at six months to 0.30 at 30 months (*p* < 0.01) - GnRH agonist: baseline 75.8 to 0.60 at six months to 4.50 at 30 months (*p* < 0.01) - No statistical difference between groups at six and 30 months VAS (sciatica): - DNG: baseline 9.50 to 4.0 at six months to 5.60 at 30 months (*p* < 0.01) - GnRH agonist: baseline 8.20 to 0.30 at six months to 1.30 at 30 months (*p* < 0.01) - No statistical difference between groups at six and 30 months	Both treatments significantly reduced pain between baseline and at six and 30 months without significant difference between the two arms
Cheewadhanaraks et al., (2012) [36]	DMPA (150 mg/12 weekly; IM) (*n* = 42)	OCP (ethinyl estradiol 0.03 mg and gestodene 0.075 mg/daily; orally) (*n* = 42)	–	VAS Verbal rating scale (VRS) (modified from the B&B Scale)	VRS (dysmenorrhoea, deep dysparaeunia and non-menstrual pain): - At 24 weeks all patients recorded a pain score of 0 at 24 weeks in all three domains for both DMPA and OCP arms VAS (dysmenorrhoea): - DMPA: median baseline score (IQR): 9 (7–10) to 0 (0–0) at 12 weeks and 0 (0–0) at 24 weeks - OCP: median baseline score (IQR): 8.2 (7–10) to 0 (0–2.8) at 12 weeks to 0 (0–3) at 24 weeks - Scores at 24 weeks were significantly higher than DMPA (*p* = 0.039) VAS (deep dysparaeunia) - DMPA: median baseline score (IQR): 3 (0–5) to 0 (0–2.2) at 12 weeks to 0 (0–2) at 24 weeks - OCP: median baseline score (IQR): 4.5 (0–7) to 0 (0–0) at 12 weeks and 0 (0–0) at 24 weeks VAS (non-menstrual pain) - DMPA: median baseline score (IQR): 2.5 (0–6.8) to 0 (0–0) at 12 weeks to 0 (0–0) at 24 weeks - OCP: median baseline score (IQR): 2 (0–6.4) to 0 (0–0.6) at 12 weeks to 0 (0–0.4) at 24 weeks	VRS and VAS scores improved significantly in both groups however DMPA was more effective than OCP at reducing dysmenorrhea scores on the VAS at 24 weeks
Crosignani et al., (2006) [37]	DMPA (104 mg/0.65 ml/3 monthly; SC) (*n* = 153)	Leuprolide (3.75 mg/monthly; orally or 11.25 mg/3 monthly; orally) (*n* = 146)	–	B&B Scale	B&B (dysmenorrhoea dysparaeunia, pelvic pain, pelvic tenderness and induration) - DMPA: mean improvement from baseline was 6.3 at six months follow-up, 6.6 at 12 months follow-up (*p* < 0.001) - Leuprolide: mean improvement from baseline was 7.3 at six months follow-up and 6.1 at 12 months follow-up (*p* < 0.001) - At six months follow-up DMPA demonstrated statistically equivalent reductions compared to leuprolide in all five of the B&B symptom domains (*p* < 0.02) - At 12 months follow-up DMPA remained statistically equivalent for four of the five symptoms; improvement of dysparaeunia was observed in both arms but was not statistically equivalent (*p* < 0.04; *p* = 0.02 required for equivalence)	DMPA was as effective as leuprolide in reducing pain associated with endometriosis
El Taha et al., (2021) [38]	Dienogest (2 mg/daily; orally) (*n* = 35)	OCP (ethinyl estradiol 0.03 mg and drospirenone 3 mg/daily; orally) drospirenone) (*n* = 35)	–	VAS B&B Scale	VAS (overall): - DNG: mean difference from baseline = 6.0 (95% CI 4.9, 7.1; *p* < 0.0001) - OCP: mean difference from baseline = 4.54 (95% CI 3.1, 5.9; *p* < 0.0001) - The difference between arms was not significant *p* = 0.111 B&B (difference between arms): - Chronic pelvic pain: *p* = 0.052 at 12 weeks, *p* = 0.526 at 24 weeks - Dysmenorrhoea: *p* = 0.521 at 12 weeks, *p* = 1 at 24 weeks - Dyspareunia: *p* = 0.376 at 12 weeks, *p* = 0.835 at 24 weeks	Both dienogest and the OCP improved endometriosis related pain, the difference between arms was not significant
Ferreira et al., (2010) [39]	LNG-IUS (*n* = 22)	Leuprolide (3.75 mg/monthly; IM) (*n* = 22)	–	VAS	VAS (overall): - LNG-IUS: mean baseline 7.3 ± 1.5 to 1.2 ± 1.75 (*p* < 0.001) - Leuprolide: mean baseline 7.1 ± 1.46 to 0.7 ± 1.37 (*p* < 0.001) - No significant difference between the two arms (*p* = 0.21)	Both LNG-IUS and leuprolide showed a significant reduction in pain after six months of treatment with no significant difference between the two
Harada et al., (2009) [40]	Dienogest (2 mg/daily; orally) (*n* = 137)	Buserelin acetate (900μg/daily; intranasally) (*n* = 134)	–	VAS Subjective symptoms during non-menstruation	VAS (overall): - DNG: mean difference ± SD from baseline to end of treatment at 24 weeks: − 30.2 ± 31.8 (lower abdominal pain) and − 15.7 ± 28.7 (lumbago) (no *p*-value) - Buserelin acetate: mean difference ± SD from baseline to end of treatment at 24 weeks: − 27.3 ± 33.8 (lower abdominal pain) and − 17.3 ± 24.8 (lumbago) (no *p*-value) Subjective symptoms during non-menstruation: - Difference of mean change between DNG and Buserelin acetate from beginning to end of treatment: − 0.32 (− 0.59, − 0.05; 95% CI; no *p*-value)	Dienogest and intranasal buserelin acetate demonstrated similar efficacy in reducing symptoms associated with endometriosis; no significant difference between the two were found
Lang et al., (2018) [41]	Dienogest (2 mg/daily; orally) (*n* = 126)	Placebo (daily; orally) (*n* = 129)	–	VAS (EAPP) B&B Scale	VAS (EAPP): - DNG: baseline mean (SD): 57.6 (20.24) to 18.9 (18.82) at 24 weeks; mean change (SD): − 38.7 (25.07) before and after treatment - Placebo: baseline mean (SD) 60.4 (21.24) to 44.7 (25.79) at 24 weeks; mean change (SD): − 15.7 (24.09) before and after treatment - DNG vs placebo: least-square mean difference in change of VAS score from baseline to 24 weeks was − 24.54 (95% CI: − 29.93 to − 19.15; *p* < 0.0001) B&B (pelvic pain) - DNG: mean difference − 2.5 (1.80) from baseline to 24 weeks - Placebo: mean difference − 0.8 (1.59) from baseline to 24 weeks B&B (physical signs score) - DNG: mean difference − 0.8 (1.32) from baseline to 24 weeks - Placebo: mean difference − 0.4 (1.29) from baseline to 24 weeks B&B (total symptom severity score) - DNG: mean difference -3.4 (2.40) from baseline to 24 weeks - Placebo: mean difference − 1.1 (2.12) from baseline to 24 weeks	Dienogest significantly reduced pain symptoms compared to placebo at 24 weeks
Margatho et al., (2020) [42]	LNG-IUS (*n* = 51)	Etonogestrel implant (ENG) (*n* = 52)	–	VAS	VAS (chronic pelvic pain): - LNG-IUS: 7.4 ± 0.2 (95% CI: 6.9, 7.9) at baseline to 4.2 ± 0.5 (95% CI: 3.2, 5.3) at 24 months (*p* < 0.001) - ENG: 7.5 ± 0.2 (95% CI: 7.1, 8.0) at baseline to 3.4 ± 0.4 (95% CI: 2.4, 4.4) at 24 months (*p* < 0.001) - No significant difference between arms at 24 months between arms (*p* = 0.211) VAS (dysmenorrhoea): - LNG-IUS: 7.4 ± 0.2 (95% CI: 6.9, 7.9), at baseline to 4.4 ± 0.5 (95% CI: 17.2, 34.9), at 24 months (*p* < 0.001) - ENG: 7.5 ± 0.1 (95% CI: 7.1, 8.0) at baseline to 4.3 ± 0.4 (95% CI: 3.3, 5.2) at 24 months (*p* < 0.001) - No significant difference between arms at 24 months (*p* = 0.88)	There was no significant difference between the effectiveness of LNG-IUS and the ENG implant for reduction of pelvic pain and dysmenorrhoea
Razzi et al., (2007) [43]	Desogestrel (75 μg/daily; orally) (*n* = 20)	OCP (Ethinyl estradiol 20 μg and Desogestrel μg mg daily; orally) (*n* = 20)	–	VAS	VAS (overall): - Desogestrel: mean end VAS was 2.5 versus 5.5 at start of treatment (*p* < 0.001) - OCP: mean end VAS was 2.3 versus 4.9 at start of treatment (*p* < 0.001)	Both interventions reduced pelvic pain significantly
Regidor et al., (2001) [44]	Lynestrenol (5 mg/twice daily; orally) (*n* = 22)	Leuprorelin acetate depot (3.75 mg/monthly; SC) (*n* = 26)	–	B&B Scale	B&B (dysmenorrhoea) - Lynestrenol: 50% (11/22) experienced reduced dysmenorrhoea - Leuprorelin: 85% (22/26) experienced reduced dysmenorrhoea B&B (chronic pelvic pain) - Lynestrenol: 59% (13/22) experienced reduced chronic pelvic pain - Leuprorelin: 69% (18/26) experienced reduced chronic pelvic pain B&B (dysparaeunia): - Lynestrenol: 23% (5/22) experienced reduced dysparaeunia - Leuprorelin: 50% (13/26) experienced reduced dysparaeunia	After six months of treatment either intervention led to a reduction in symptoms, however, leuprorelin was more effective overall
Schlaff et al., (2006) [45]	DMPA (104 mg/0.65 mL/3 monthly; SC) (*n* = 136)	Leuprolide (11.25 mg/3 monthly; IM) (*n* = 138)	–	B&B Scale	B&B (dysmenorrhea, dyspareunia, pelvic pain, pelvic tenderness, and induration) - DMPA: mean change from baseline was − 6.2 at 6 months and − 5.3 at 18 months (*p* < 0.001) - Leuprolide: mean change from baseline was − 7.7 at 6 months and − 5.1 at 18 months (*p* < 0.001) - Treatment with DMPA was statistically equivalent (*p* < 0.02) to treatment with leuprolide for four out of five symptoms at 6 months and for all symptoms at 18 months	DMPA and leuprolide were equally effective at reducing pain related symptoms associated with endometriosis
Strowitzki et al., (2010) [46]	Dienogest (2 mg/daily; orally) (*n* = 102)	Placebo (daily; orally) (*n* = 96)	–	VAS (EAPP) B&B Scale	VAS (EAPP): - Dienogest vs placebo: significantly superior reducing EAPP in both the full analysis set (*p* = 0.00165) and the per-protocol set (*p* = 0.00007) - Full analysis set: o Dienogest (*n* = 102): mean reduction of 27.4 o Placebo (*n* = 96): mean reduction of 15.1 o Dienogest versus placebo: mean group difference: 12.3 (95% CI: 6.4, 18.1; *p* < 0.0001) - Per-protocol set: o Dienogest (*n* = 74) versus placebo (*n* = 70): mean group difference: 13.2 (95% CI: 7.3, 19.2; *p* < 0.0001) Change in intake of supportive analgesia: - Dienogest: decrease of 4.4 ± 6.4 tablets/28 days - Placebo: decrease of 3.7 ± 8.2 tablets/28 days - Mean group difference: 0.74 tablets/28 days; (95% CI: − 1.412, 2.895; no p-value) B&B (pelvic pain, dysmenorrhoea, pelvic tenderness and dysparaeunia): - At study end, the proportion of patients in the ‘‘none’’ category in the dienogest and placebo groups, respectively, were 11.8% versus 2.1% for ‘‘pelvic pain’’, 17.6% versus 11.5% for ‘‘physical signs’’, and 7.8% versus 2.1% for ‘‘total symptom and sign severity.’’	Dienogest was clinically superior in reducing VAS scores by week 12 compared to placebo but not in changes to intake of supportive analgesia
Tanmahasamut et al., (2012) [47]	LNG-IUS (*n* = 28)	Expectant management (*n* = 27)	–	VAS	VAS (dysmenorrhoea): - LNG-IUS (*n* = 28): Baseline median score (IQR): 87 (77.5–100) to 4.5 (0.0–11.5) at 12 months; median reduction (IQR): 81.0 (51.5–87.5; *p* < 0.001) - Expectant management (*n* = 26): Baseline median score (IQR): 90 (75–95) to 23 (7–65) at 12 months; median reduction (IQR): 50 (0.0–78.0; *p* < 0.001) - LNG-IUS vs Expectant management: *p* = 0.006 VAS (non-cyclic pelvic pain): - LNG-IUS (*n* = 28): Baseline median score (IQR): 42 (22.0–82.5) to 0.0 (0.0–0.0) at 12 months; median reduction (IQR): 48.5 (19.5–84.25; *p* < 0.001) - Expectant management (*n* = 19): Baseline median score (IQR): 37.5 (26.25–58.75) to 5.0 (0.0–39.75) at 12 months; median reduction (IQR): 22 (1.5–46.5; *p* = 0.031) - LNG-IUS vs Expectant management: *p* = 0.038 VAS (dysparaeunia): - LNG-IUS (*n* = 12): Baseline median score (IQR): 25.0 (10.0–50.0) to 0.0 (0.0–5.5) at 12 months; median reduction (IQR): 15.0 (4.0–38.5; *p* = 0.023) - Expectant management (*n* = 7): Baseline median score (IQR): 43.0 (16.0–91.75) to 3.0 (0.0–100.0) at 12 months; median reduction (IQR): 19.0 (21.0–66.75); *p* = 0.345 - LNG-IUS vs Expectant management: *p* = 0.831	LNG-IUS significantly reduced dysmenorrhea and non-cyclic pelvic pain but not dysparaeunia compared to the non-intervention group
Tanmahasamut et al., (2017) [48]	Desogestrel (0.075 mg/daily; orally) (*n* = 20)	Placebo (daily; orally) (*n* = 20)	–	VAS	VAS (overall): - Intention-to-treat: o Desogestrel (*n* = 20): median score change (range): − 84.00 (− 100, 19) o Placebo (*n* = 20): median score change (range): − 57.00 (− 100, 0) o Intergroup comparison: *p* = 0.005 - Per protocol: o Desogestrel (*n* = 19): median score change (range): − 85 (− 100, − 50) o Placebo (*n* = 19): median score change (range): − 58 (− 100, − 18) o Intergroup comparison: *p* = 0.003 VAS (dysmenorrhoea): - Intention-to-treat: o Desogestrel (*n* = 20): median score change (range): − 84 (− 100, 19) o Placebo (*n* = 20): median score change (range): − 61 (− 96, 0) o Intergroup comparison: *p* = 0.005 - Per-protocol: o Desogestrel (*n* = 19): median score change (range): − 84 (− 100, − 29) o Placebo (*n* = 19): median score change (range): − 61 (− 96, − 18) o Intergroup comparison: *p* = 0.002 VAS (non-cyclic pelvic pain): - Intention-to-treat: o Desogestrel (*n* = 18): median score change (range): − 81 (− 100, 23) o Placebo (*n* = 18): median score change (range): − 51 (− 100, 35) o Intergroup comparison: *p* = 0.007 - Per protocol: o Desogestrel (*n* = 17): median score change (range): − 81 (− 100, − 12) o Placebo (*n* = 17): median score change (range): − 52 (− 100, 35) o Intergroup comparison: *p* = 0.004 VAS (dysparaeunia): - Intention-to-treat: o Desogestrel (*n* = 7) median score change (range): -59 (− 91, 22) o Placebo (*n* = 11) median score change (range): − 51 (− 84, 13) o Intergroup comparison: *p* = 0.342 - Per protocol: o Desogestrel (*n* = 6) median score change (range): − 66 (− 91, − 46) o Placebo (*n* = 10) median score change (range): − 52 (− 64, 13) o Intergroup comparison: *p* = 0.159 Number of rescue medications (tablets): - Acetaminophen: o Desogestrel (*n* = 20) median score change (range): 16 (0, 72) o Placebo (*n* = 20) median score change (range): 17 (0, 120) o Intergroup comparison *p* = 0.659 - Mefenamic acid: o Desogestrel (*n* = 20) median score change (range): 5 (0, 44) o Placebo (*n* = 20) median score change (range): 15 (0, 93) o Intergroup comparison *p* = 0.036	Desogestrel significantly lowered overall pain, non-cyclic pelvic pain and dysmenorrhoea but not dysparaeunia compared to placebo
Vercellini et al., (2005) [49]	Norethindrone acetate (2.5 mg/daily; orally) (*n* = 45)	OCP (Ethinyl estradiol 0.01 mg and cyproterone acetate 3 mg/daily; orally) (*n* = 45)	–	VAS VRS	VAS (dyschezia): - Norethindrone acetate (*n* = 22): mean decrease at 12 months 45.7 ± 21.8 (*p* < 0.05) - OCP (*n* = 14): mean decrease at 12 months 42.9 ± 22.0 (*p* < 0.05) - Intergroup comparison: *p* > 0.05 VAS (non-menstrual pain): - Norethindrone acetate (*n* = 20): mean decrease at 12 months 43.0 ± 21.7 (*p* < 0.05) - OCP (*n* = 18): mean decrease at 12 months 27.5 ± 31.2 (*p* < 0.05) - Intergroup comparison: *p* > 0.05 VAS (deep dysparaeunia): - Norethindrone acetate (*n* = 25): mean decrease at 12 months 35.6 ± 28.3 (*p* < 0.05) - OCP (*n* = 23): mean decrease at 12 months 35.6 ± 28.3 (*p* < 0.05) - Intergroup comparison: *p* > 0.05 VAS (dysmenorrhoea): - Norethindrone acetate (*n* = 37): mean decrease at 12 months 72.8 ± 22 (*p* < 0.05) - OCP (*n* = 34): mean decrease at 12 months 63.7 ± 23.3 (*p* < 0.05) - Intergroup comparison: *p* > 0.05 VRS (dyschezia): - Norethindrone acetate (*n* = 22): mean decrease at 12 months 1.5 ± 0.7 - OCP (*n* = 14): mean decrease at 12 months 1.4 ± 0.6 - Intergroup comparison: *p* > 0.05 VRS (non-menstrual pain): - Norethindrone acetate (*n* = 20): mean decrease at 12 months 1.4 ± 0.6 (*p* < 0.05) - OCP (*n* = 18): mean decrease at 12 months 0.9 ± 0.9 (*p* < 0.05) - Intergroup comparison: *p* > 0.05 VRS (deep dysparaeunia): - Norethindrone acetate (*n* = 25): mean decrease at 12 months 1.2 ± 0.8 (*p* < 0.05) - OCP (*n* = 23): mean decrease at 12 months 1.2 ± 0.8 (*p* < 0.05) - Intergroup comparison: *p* > 0.05 VRS (dysmenorrhoea): - Norethindrone acetate (*n* = 37): mean decrease at 12 months 2.4 ± 0.8 (*p* < 0.05) - OCP (*n* = 34): mean decrease at 12 months 2.1 ± 0.8 (*p* < 0.05) - Intergroup comparison: *p* > 0.05	At the end of 12 months treatment with both intervention arms had significantly reduced pain with no significant differences between arms
Fibroids						
Verspyck et al., (2000) [50]	Lynestrenol (5 mg/twice daily; orally) (*n* = 23)	Leuprorelin (3.75 mg/monthly; IM) (*n* = 33)	–	VAS	VAS (overall): - Lynestrenol: 4.05 at baseline to 2.10 at day 28 to 2.20 at week 16 - Leuprorelin: 4.86 at baseline to 3.06 at day 28 to 1.36 at week 16	Both interventions significantly improved symptoms and reduced pain with no significant differences between the two arms
Whitaker et al., (2023) [51]	LNG-IUS (*n* = 38)	UPA (5 mg/daily for 3, 12-week cycles separated by 4-week treatment free intervals/orally) (*n* = 43)	–	Uterine Fibroid Symptom and Quality of Life Questionnaire (UFS-QOL)	UFS-QOL (symptom domain): - LNG-IUS (*n* = 27): 57.4 at baseline to 33.1 (*n* = 17) at 12 months - UPA (*n* = 30): 53.1 at baseline to 26.0 (*n* = 23) at 12 months - Mean difference: 6.0 (− 10.6, 22.5)	At 12 months both interventions improved fibroid symptoms (UFS-QOL) but there was no significant difference between the interventions

### Endometriosis

19 studies [31–49] evaluated the efficacy of progestins for the management of endometriosis-associated pain. In 14 studies [31–35, 37–40, 42–45, 49], progestins were found to be equivalent to comparator interventions for reducing pain associated with endometriosis. Five studies [36, 41, 46–48] found progestins to be significantly more efficacious than comparator interventions (placebo (*n* = 2), expectant management (*n* = 1) and an OCP (*n* = 1)) for reducing pain associated with endometriosis.

A 2012 study [47] conducted in Thailand compared the efficacy of the LNG-IUS to expectant management in reducing dysmenorrhoea, non-cyclic pelvic pain and dysparaeunia associated with endometriosis after 12 months. This study identified that compared to expectant management (*n* = 27), LNG-IUS (*n* = 28) significantly reduced dysmenorrhoea (*p* = 0.006) and non-cyclic pelvic pain (*p* = 0.038), but not dysparaeunia (*p* = 0.831). A second 2012 study [36] from Thailand found that depot medroxyprogesterone acetate (DMPA) (*n* = 42) was more effective than an OCP (*n* = 42) in reducing dysmenorrhoea (*p* = 0.039), but not other pain symptoms associated with endometriosis at 24 weeks.

Two studies [41, 46] comparing daily dienogest to placebo found dienogest to significantly reduce pain compared to placebo. One of these, a 2018 trial [41] conducted in China, found that women who received dienogest daily (*n* = 126) compared to placebo (*n* = 129) experienced significantly reduced endometriosis-associated pelvic pain (EAPP). This study identified a mean reduction in VAS scores, representing EAPP, between the two arms of 24.54 (*p* < 0.0001) at 24 weeks; reflecting the greater efficacy of dienogest. Concurrently, a 2010 multi-centre trial [46] (*n* = 33) conducted in Germany, Italy, and Ukraine compared the efficacy of dienogest (*n* = 102) versus placebo (*n* = 96) in reducing EAPP. VAS score reductions were significantly greater among women in the dienogest arm who, on average, experienced a VAS score reduction of 12.3 (*p* < 0.0001) more than those in the placebo arm.

Finally, a 2017 study [48] conducted in Thailand found that women who received daily desogestrel (*n* = 20) compared to placebo (*n* = 20) experienced significantly reduced dysmenorrhoea (*p* = 0.005), non-cyclic pelvic pain (*p* = 0.007) and overall pain (*p* = 0.005), but no difference in dysparaeunia (*p* = 0.342). Additionally, the number of mefenamic acid tablets consumed by those receiving desogestrel was significantly lower than those receiving placebo (*p* = 0.036), however, there was no significant difference in acetaminophen intake between the two arms of the study (*p* = 0.659).

### Fibroids

Two studies [50, 51] evaluated the efficacy of progestins in reducing pain associated with fibroids. In each of these studies, no significant difference between progestins and comparator interventions for the reduction of fibroid-related pain was identified. However, both studies were found to be at a high risk of bias, therefore, their findings should be interpreted with caution.

One study [50], a multi-centre RCT conducted in France in 2000, compared lynestrenol to leuprorelin (a GnRH agonist). This study found that the women receiving lynestrenol (*n* = 23) experienced a reduction in pelvic pain as measured by the VAS from 4.05 at baseline to 2.20 at week 16. Meanwhile, women who received leuprorelin (*n* = 33) recorded a reduction in VAS of 4.86 at baseline to 1.36 at week 16. The authors concluded that both interventions significantly reduced pain with no significant differences between study groups.

A 2023 study [51] conducted in the UK compared the efficacy of LNG-IUS to UPA; a selective progesterone receptor modulator. Among women with fibroids, those receiving LNG-IUS (*n* = 38) experienced a greater reduction of, on average, six points on the UFS-QOL compared to those receiving UPA (*n* = 43); however, this was not statistically significant.

### Adverse effects

There was a diverse range of adverse effects recorded by participants of the included studies, with differences between those treated with progestin and comparator interventions (Table 3). The most common adverse effect encountered by women receiving a progestin therapy was spotting/irregular bleeding; mentioned in 12 studies [31, 33–38, 40, 41, 43, 45, 48] with incidence ranging between 5.4% and 95%. Four of these studies [35, 37, 45, 48] stated the rate of spotting/irregular bleeding to be significantly higher among women receiving progestins versus comparator interventions, in one study [38] this was reversed and spotting/irregular bleeding was significantly more common in the comparator arm whilst in the remaining seven studies [31, 33, 34, 36, 40, 41, 43] statistical significance was not mentioned. Comparatively, the most common adverse effect for women receiving comparator interventions was hot flushes. Seven studies [31, 35–37, 40, 45, 50] mentioned this, notably in all except one the comparator intervention was a GnRH agonist. Overall, the incidence of hot flushes in these studies ranged between 11.1% and 86.4%, with three studies [35, 37, 45] demonstrating a significantly higher rate among women receiving comparator interventions. In the remaining four studies [31, 36, 40, 50] statistical significance was not stated. Other frequently recorded adverse effects common to both arms were headache, nausea, breast tenderness and weight gain.

**Table 3:** Adverse effects

Author (Year)	Adverse effects (Progestin)	Adverse effects (Comparator)	Additional comments
Endometriosis			
Bayoglu Tekin et al., (2011) [31]	*N* = 20 Irregular bleeding: 13 (65%), abdominal pain: 8 (40%), weight gain: 2 (10%), ovarian cysts: 11 (55%), amenorrhoea: 0 (0%), vasomotor symptoms: 0 (0%)	*N* = 20 Irregular bleeding: 0 (0%), abdominal pain: 0 (0%), weight gain: 1 (5%), amenorrhoea: 6 (30%), vasomotor symptoms: 10 (50%), ovarian cysts: 0 (0%)	–
Carr et al., (2014) [32]	*N* = 83 Any AE: 75 (89.3%), headache: 15 (17.9%), nausea: 13 (15.5%), nasopharyngitis: 9 (10.7%), upper respiratory tract infection: 10 (11.9%), sinusitis: 6 (7.1%), pharyngolaryngeal pain: 3 (3.6%), mood swings: 10 (11.9%), influenza: 2 (2.4%), acne: 7 (8.3%), back pain: 4 (4.8%), anxiety: 4 (4.8%), urinary tract infection: 5 (6.0%), vaginal mycosis: 3 (3.6%), fatigue: 6 (7.1%), ovarian cyst: 1 (1.2%), diarrhoea: 8 (9.5%), arthralgia: 2 (2.4%), insomnia: 4 (4.8%), nasal congestion: 3 (3.6%), migraine: 5 (6.0%), dizziness: 8 (9.5%), depression: 4 (4.8%), sinus congestion: 5 (6.0%), cough: 3 (3.6%), abdominal distension: 6 (7.1%), dyspepsia: 3 (3.6%)	Elagolix 150 mg: *N* = 84 Any AE: 72 (85.7%), headache: 22 (26.2%), nausea: 16 (19.0%), nasopharyngitis: 9 (10.7%), upper respiratory tract infection: 8 (9.5%), sinusitis: 7 (8.3%), pharyngolaryngeal pain: 7 (8.3%), mood swings: 7 (8.3%), influenza: 7 (8.3%), acne: 7 (8.3%), back pain: 6 (7.1%), anxiety: 6 (7.1%), urinary tract infection: 5 (6.0%), vaginal mycosis: 5 (6.0%), fatigue: 5 (6.0%), ovarian cyst: 5 (6.0%), diarrhoea: 4 (4.8%), arthralgia: 4 (4.8%), insomnia: 4 (4.8%), nasal congestion: 4 (4.8%), migraine: 4 (4.8%), dizziness: 3 (3.6%), depression: 3 (3.6%), sinus congestion: 3 (3.6%), cough: 2 (2.4%), abdominal distension: 2 (2.4%), dyspepsia: 1 (1.2%) Elagolix 75 mg: *N* = 84 Any AE: 74 (88.1%), headache: 23 (27.4%), nausea: 13 (15.5%), nasopharyngitis: 18 (21.4%), upper respiratory tract infection: 10 (11.9%), sinusitis: 7 (8.3%), pharyngolaryngeal pain: 7 (8.3%), mood swings: 6 (7.1%), influenza: 5 (6.0%), acne: 2 (2.4%), back pain: 10 (11.9%), anxiety: 4 (4.8%), urinary tract infection: 8 (9.5%), vaginal mycosis: 4 (4.8%), fatigue: 3 (3.6%), ovarian cyst: 0 (0.0%), diarrhoea: 9 (10.7%), arthralgia: 9 (10.7%), insomnia: 7 (8.3%), nasal congestion: 6 (7.1%), migraine: 4 (4.8%), dizziness: 6 (7.1%), depression: 6 (7.1%), sinus congestion: 5 (6.0%), cough: 6 (7.1%), abdominal distension: 0 (0.0%), dyspepsia: 5 (6.0%)	–
Caruso et al., (2022) [33]	*N* = 98 Spotting: 17 (18.4%), nausea: 11 (11.9%), breast tenderness: 15 (16.3%)	*N* = 99 Spotting: 16 (17.9%), nausea: 12 (13.5%), breast tenderness: 13 (14.6%)	–
Carvalho et al., (2018) [34]	*N* = 45 (90 days) Spotting: 36.1%, prolonged bleeding: 21.6% *N* = 40 (180 days) Infrequent bleeding: 30% and spotting: 22.1%	*N* = 50 (90 days) Infrequent bleeding: 30% and spotting: 22.1% *N* = 45 (180 days) Amenorrhoea: 28.8% and infrequent bleeding: 24.4%	–
Ceccaroni et al., (2021) [35]	*N* = 65 Amenorrhea: 52 (80%), spotting: 18 (27.7%), hot flushes: 8 (12%), headache: 15 (23%), swelling: 21 (32%), breast tenderness: 5 (7.7%), alopecia: 10 (15%), vaginal dryness: 13 (20%), decreased libido: 7 (10.8%), mood disorders: 23 (35.4%)	*N* = 81 Amenorrhea: 77 (95%), spotting: 1 (1.2%), hot flushes: 70 (86.4%), headache: 12 (14.8%), swelling: 20 (24.7%), breast tenderness: 2 (2.4%), alopecia: 17 (21%), vaginal dryness: 13 (16%), decreased libido: 10 (16%), mood disorders: 35 (43%)	Spotting significantly more common in the progestin arm (*p* < 0.001) Hot flushes significantly more common in the comparator arm (*p* < 0.001)
Cheewadhanaraks et al., (2012) [36]	*N* = 39 Oily skin: 15 (38.5%), irritability: 12 (30.8%), amenorrhoea: 7 (17.9%), spotting: 28 (71.8%), breakthrough bleeding: 4 (10.3%)	*N* = 42 Mastalgia: 21 (50%), nausea: 15 (35.7%), amenorrhoea: 3 (7.9%), spotting: 24 (63.2%), breakthrough bleeding: 11 (28.9%)	–
Crosignani et al., (2006) [37]	*N* = 152 Nausea: 17 (11.2%), headache: 5 (3.3%), breast pain: 8 (5.3%), intermenstrual bleeding: 19 (12.5%), hot flushes: 9 (5.9%)	*N* = 143 Nausea: 10 (7%), headache: 9 (6.3%), breast pain: 5 (3.5%), intermenstrual bleeding: 1 (0.7%), hot flushes: 24 (16.8%)	Patients receiving DMPA experienced significantly more intermenstrual bleeding, uterine haemorrhage and vaginal haemorrhage (*p* ≤ 0.05) Patients receiving leuprolide reported significantly more hot flushes (*p* = 0.03)
El Taha et al., (2021) [38]	*N* = 31 Headache: 10 (32.3%), breast pain: 6 (19.4%), sleep disorder: 3 (9.7%), decreased libido: 1 (3.2%), fatigue: 3 (9.7%), nausea/vomiting: 5 (16.1%), mood swings: 14 (45.2%), abdominal discomfort/bloating: 5 (16.1%), weight gain: 3 (9.7%), abnormal uterine bleeding: 21 (67.7%)	*N* = 32 Headache: 19 (59.4%), breast pain: 15 (46.9%), sleep disorder: 9 (28.1%), decreased libido: 3 (9.4%), fatigue: 9 (28.1%), nausea/vomiting: 17 (53.1%), mood swings: 24 (75%), abdominal discomfort/bloating: 12 (37.5%), weight gain: 11 (34.4%), abnormal uterine bleeding: 29 (90.6%)	All significantly less frequent among those receiving dienogest except decreased libido and abdominal discomfort/bloating for which there was no significant difference between the two arms
Ferreira et al., (2010) [39]	None stated	None stated	–
Harada et al., (2009) [40]	*N* = 129 Genital bleeding: 122 (95%), hot flushes: 64 (50%), headache: 32 (25%)	*N* = 126 Genital bleeding: 85 (67%), hot flushes: 85 (67%), headache 43 (34%)	–
Lang et al., (2018) [41]	*N* = 126 Any AEs: 63 (50%), study drug related AEs: 37 (29.4%), vaginal haemorrhage: 10 (7.9%)	*N* = 129 Any AE: 57 (44.2%), study drug related AEs: 13 (10.1%), vaginal haemorrhage: 3 (2.3%)	–
Margatho et al., (2020) [42]	None stated	None stated	
Razzi et al., (2007) [43]	*N* = 20 Breakthrough bleeding: 4 (25%)	*N* = 20 Weight gain: 3 (15%)	–
Regidor et al., (2001) [44]	*N* = 26 Hot flushes: 21 (80.8%), sweating: 14 (53.8%), nausea: 2 (7.7%), body weight gain: 2 (7.7%), headache: 2 (7.7%), psychological alterations: 3 (11.5%), allergic reactions: 1 (3.8%), tiredness: 1 (3.8%), acne: 0 (0%)	*N* = 22 Hot flushes: 13 (59.1%), sweating: 9 (40.9%), nausea: 0 (0%), body weight gain: 3 (13.6%), headache: 0 (0%), psychological alterations: 1 (4.5%), allergic reactions: 0 (0%), tiredness: 0 (0%), acne: 5 (22.7%)	–
Schlaff et al., (2006) [45]	*N* = 130 Any AE: 113 (86.9%), injection-site reaction: 9 (6.9%), headache: 10 (7.7%), insomnia: 3 (2.3%), libido decrease: 3 (2.3%), intermenstrual bleeding: 7 (5.4%), hot flushes: 3 (2.3%)	*N* = 115 Any AE: 115 (85.2%), injection-site reaction: 0 (0%) headache: 14 (10.4%), insomnia: 7 (5.2%), libido decrease: 7 (5.2%), intermenstrual bleeding: 1 (0.7%), hot flushes: 15 (11.1%)	Injection-site reactions and intermenstrual bleeding were significantly more common in the DMPA group (*p* < 0.05) Hot flushes were significantly more common in the leuprolide group (*p* < 0.05)
Strowitzki et al., (2010) [46]	*N* = 102 Headache: 11 (10.8%), cystitis: 3 (2.9%), nausea: 3 (2.9%), nasopharyngitis: 2 (1.9%), bronchitis: 2 (1.9%), influenza: 2 (1.9%), depression: 2 (1.9%), breast discomfort: 2 (1.9%), vomiting: 0 (0%), gastritis: 0 (0%), proteinuria: 0 (0%), vaginal candidiasis: 0 (0%), asthenia: 2 (1.9%)	*N* = 96 Headache: 5 (5.2%), cystitis: 0 (0%), nausea: 1 (1.0%), nasopharyngitis: 6 (6.3%), bronchitis: 3 (3.1%), influenza: 3 (3.1%), depression: 2 (2.1%), breast discomfort: 1 (1.0%), vomiting: 3 (3.1%), gastritis: 2 (2.1%), proteinuria: 2 (2.1%), vaginal candidiasis: 2 (2.1%), asthenia: 0 (0%)	–
Tanmahasamut et al., (2012) [47]	*N* = 27 Bloating: 10 (37.0%), acne: 16 (59.3%), oily skin: 20 (74.1%), melasma: 6 (22.2%), weight gain: 17 (63.0%), breast tenderness: 18 (66.7%), headache: 13 (48.1%), nausea: 11 (40.7%), leukorrhea: 1 (3.7%)	*N* = 23 Bloating: 16 (69.6%), acne: 13 (56.5%), oily skin: 16 (69.6%), melasma: 0 (0%), weight gain: 13 (56.5%), breast tenderness: 9 (39.1%), headache: 17 (73.9%), nausea: 9 (39.1%), leukorrhea: 3 (13.0%)	Bloating was significantly more common among those receiving expectant care (*p* = 0.021) Melasma was significantly more common among those receiving LNG-IUS (*p* = 0.015) There was no significant difference between the arms for incidence of the other adverse effects
Tanmahasamut et al., (2017) [48]	*N* = 19 Amenorrhoea: 7 (36.8%), spotting: 8 (42.1%), light bleeding: 1 (5.3%), acne: 13 (68.4%), breast pain: 10 (52.6%), headache: 8 (42.1%), nausea/vomiting: 4 (21.1%), hair loss: 4 (21.1%), mood change: 3 (15.8%), and rash: 1 (5.3%)	*N* = 19 Amenorrhoea: 0 (0%), spotting: 2 (10.5%), light bleeding: 0 (0%), acne: 9 (47.4%), breast pain: 9 (47.4%), headache: 8 (42.1%), nausea/vomiting: 3 (15.8%), hair loss: 3 (15.8%), mood change: 1 (5.3%), and rash: 2 (10.5%)	Menstruation alteration (amenorrhoea, spotting and light bleeding) were significantly more common among those in the desogestrel arm (*p* < 0.001)
Vercellini et al., (2005) [49]	*N* = 42 Any AE: 21 (50%), weight gain: 12 (28.6%), headache: 2 (4.8%), nausea: 0 (0%), depression: 3 (7.1%), decreased libido: 4 (9.5%), acne: 2 (4.8%), bloating: 1 (2.4%), swelling: 4 (9.5%), breast tenderness: 0 (0%), hypertriglyceridemia: 0 (0%), erythematous cutaneous reaction: 1 (2.4%)	*N* = 41 Any AE: 16 (39%), weight gain: 7 (17.1%), headache: 3 (7.3%), nausea: 3 (7.3%), depression: 2 (4.9%), decreased libido: 2 (4.9%), acne: 1 (2.4%), bloating/swelling: 1 (2.4%), breast tenderness: 1 (2.4%), hypertriglyceridemia: 1 (2.4%), erythematous cutaneous reaction: 0 (0%)	There was no significant difference in overall prevalence of AEs between arms (*p* = 0.43)
Fibroids			
Verspyck et al., (2000) [50]	*N* = 23 Hot flushes: 4 (17.4%), headache: 2 (8.7%), nausea: 4 (17.4%), weight gain: 3 (13.0%), oedema: 1 (4.3%), sleep disorder: 1 (4.3%), mood disorder: 2 (8.7%), anxiety: 1 (4.3%), vaginal dryness: 0 (0%), cutaneous disorders: 2 (8.7%)	*N* = 33 Hot flushes: 19 (57.6%), headache: 11 (33.3%), nausea: 2 (6.1%), weight gain: 2 (6.1%), oedema: 3 (9.1%), sleep disorder: 3 (9.1%), mood disorder: 1 (3.0%), anxiety: 1 (3.0%), vaginal dryness: 4 (12.1%), cutaneous disorders: 2 (6.1%)	The frequency of adverse events did not differ significantly between the two groups
Whitaker et al., (2023) [51]	Not separated for patients with fibroids	Not separated for patients with fibroids	–

### Bone mineral density

Five studies [32, 37, 40, 41, 45] included an analysis of the impact of treatment with progestins and comparator interventions on BMD. In two studies [32, 41] neither progestins nor comparator interventions caused significant reductions in BMD. In three studies [37, 40, 45] progestin use was associated with statistically significant reductions in BMD, ranging from − 1.0 to − 0.50%. Notably, in two of these [37, 45], changes in BMD were no longer statistically significant after 12 months of follow-up. Further, across all three studies where BMD fell, progestins caused significantly lower reductions in BMD compared to GnRH agonist comparators.

## Discussion

### Main findings

To our knowledge, this is the first review to compile evidence from RCTs assessing the analgesic efficacy of progestins across multiple gynaecological conditions. This review collated and compared 21 RCTs representing a total of 1317 women treated with progestins and 1428 treated with comparator interventions evaluating the analgesic efficacy in the context of endometriosis and fibroids. The 19 trials concerning endometriosis suggest that progestins are efficacious in reducing pain, with all but one showing a significant reduction in pain levels overall. Further, there were five studies where progestins were superior compared to other therapies in reducing pain associated with endometriosis. Limited and low-quality evidence examining the efficacy of progestins for the management of pain associated with fibroids was identified. The two included trials suggest that progestins reduced pain, however, neither found a significant difference between progestins and comparator interventions for doing so. Concerning adverse effects, spotting/irregular bleeding was more common among those receiving progestins whilst hot flushes were the main side effect experienced by those receiving GnRH agonists. Of the five studies that assessed treatment impacts on BMD, three found that progestins significantly reduced BMD. Notably, in all three instances these impacts were significantly less than those produced by comparator interventions and in two of the three studies where reductions in BMD were observed, reductions were no longer statistically significant after 12 months of follow-up.

### Comparison to existing evidence

Our review builds upon the findings of a 2022 meta-analysis that found progestins significantly improved pain symptoms associated with endometriosis [28]. Notably, this meta-analysis relied heavily on data from observational studies, identifying only four eligible RCTs. Our review identified several new RCTs published since the completion of a 2019 systematic review that assessed the efficacy of hormonal contraception, including progestin therapies, on outcomes in endometriosis [27]. Both reviews echo our findings that progestins are efficacious as analgesics in the setting of endometriosis. We were able to expand upon these findings to a greater range of progestins using data from recent RCTs only. There are also data in the literature that conflict with these findings. For example, a 2012 Cochrane review identified 13 RCTs relating to the effectiveness of progestagens and anti-progestagens for reducing pain associated with endometriosis. Ultimately that review concluded there was only limited evidence available to suggest progestagens and anti-progestagens were effective in achieving pain reduction [52].

While both studies [50, 51] relating to progestin use for management of fibroid-related pain determined that they were effective in reducing fibroid-related pain, both were found to be at a high risk of bias and neither were able to determine whether progestins were more efficacious than comparator interventions. These inconclusive findings are mirrored by the findings of a 2020 Cochrane review of four studies which examined the role of progestogens and progestogen-releasing intrauterine systems for the treatment of uterine fibroids [53]. Ultimately, that review was unable to draw conclusions about the efficacy of progestins for managing fibroids and fibroid-related pain due to very low study quality and methodological flaws [53].

The finding of our review that the negative impacts on BMD caused by progestins are short lived is consistent with the results of a 2005 systematic review of 39 studies [54]. That review identified that women receiving DMPA had lower BMD than those who did not, however, that these reductions were within one standard deviation and recovered spontaneously after discontinuation [54]. Additionally, our review illustrated that compared to GnRH agonists, impacts on BMD caused by progestins were lower. This finding is mirrored in a 2014 systematic review of four RCTs which found that GnRH agonists produced greater reductions in BMD compared to progestogens [55].

### Strengths and weaknesses

Strengths of this review include well-defined eligibility criteria and a comprehensive search strategy, enabling the detection of contemporary and relevant RCTs in keeping with our research question. Compared to similar reviews, we compiled data from RCTs only, therefore, minimising bias associated with using data from observational studies. The wide scope of this review is a strength; we included a variety of progestins, routes of administration, doses and treatment durations, and we consider these data valuable in understanding the efficacy of different progestin therapies in various contexts. Concurrently, however, the heterogeneity of included studies made it difficult to draw definitive conclusions and at times to make meaningful comparisons across studies. Another weakness of this review was the dearth of evidence pertaining to fibroids and PMS, limiting the generalisability of our findings to these conditions and limiting our ability to comprehensively answer our initial research question. The execution of the search strategy across only two databases may mean we missed potentially relevant articles. Importantly, the representativeness of our findings may also be undermined by our search strategy which did not specifically seek out relevant articles that included trans and gender-diverse individuals with uteri. Our decision to include studies deemed to be at a high risk of bias may impinge on the robustness of our findings, especially those relating to fibroids. Concerning the geographic distribution of included studies, most were conducted in high-income countries with limited representation from low- and middle-income settings, therefore potentially limiting the applicability of our findings to these contexts. As we did not conduct a meta-analysis, quantitative assessment of the pooled effectiveness of progestin therapies for pain management was not performed.

### Implications on practice

The added value of this review is its comparison of the analgesic efficacy of progestins across multiple conditions. This review reiterates the efficacy of progestins in reducing endometriosis-related pain, affirming their viability as alternative analgesics with a lower thrombosis risk to an OCP. To a lesser extent, this review suggests that progestins may be effective in reducing fibroid-related pain, however, more data are needed to demonstrate this. This review also highlighted that various types and administration routes of progestins were effective as analgesics, underscoring their versatility and potential to be personalised to unique patient needs. In terms of impact on BMD, compared to GnRH agonists, this review demonstrated that progestins might be less deleterious to BMD, indicating their potential utility as safer long-term analgesics in patients at high risk of BMD loss and associated sequelae.

### Future research

The inability of this review to identify any contemporary studies investigating the efficacy of progestins for the management of PMS-related pain indicates an area of future investigation. Concurrently, the very limited representation of studies relating to fibroid-related pain warrants further investigation and robust research. Further studies evaluating the existence of a dose–response relationship across all three conditions would be valuable. Finally, the lack of studies comparing surgical and medical therapies for the management of endometriosis-related pain included in our review was reflected in other studies, indicating the need for further research in this space [15].

## Conclusion

Our findings demonstrate evidence for the efficacy of progestins in the management of pain associated with endometriosis and limited data suggesting they may be efficacious in reducing pain associated with fibroids. Further robust research and data are required to confirm whether progestins are efficacious in reducing pain associated with fibroids and PMS, and to establish to what extent dose–response relationships may play in maximising analgesic efficacy across all three conditions.

## Supplementary Information

Below is the link to the electronic supplementary material.

Supplementary file1 (DOCX 24 KB)

Supplementary file2 (DOCX 18 KB)

Supplementary file3 (DOCX 19 KB)

Supplementary file4 (DOCX 491 KB)

## Data Availability

Complete data extracted from included studies not published in this review and supporting documents are available upon request from the corresponding author.
