# Supplementary material for: The efficacy of progestins in managing pain associated with endometriosis, fibroids and pre-menstrual syndrome: a systematic review
Source: Arch Gynecol Obstet. 2025 Mar 11;311(6):1511–33. doi: 10.1007/s00404-025-07957-0 (PMC12055938; doi:10.1007/s00404-025-07957-0)
Supplement: Supplementary file 2 — Supplementary file2 (DOCX 18 KB) [file 404_2025_7957_MOESM2_ESM.docx]

**Online Resource 2: Search Strategy**

**The Efficacy of Progestins in Managing Pain Associated with Endometriosis, Fibroids and Pre-Menstrual Syndrome: A Systematic Review**

**Authors:** Connor Luke Allen^1, 2^. Saikat Banerjee^3^. Mahantesh Karoshi^4^. Peter Humaidan^5, 6^ . Farshad Tahmasebi^4^.

^1^Department of Medicine, Nursing and Health Sciences, Monash University, Melbourne, Australia

^2^Western Health, Melbourne, Australia

^3^CEES-u: Cambridge University Hospitals, Cambridge, United Kingdom

^4^Royal Free London NHS Foundation Trust, London, United Kingdom

^5^The Fertility Clinic, Skive Regional Hospital, Skive, Denmark

^6^Department of Clinical Medicine, Aarhus University, Denmark

Corresponding author:

Dr Connor Luke Allen

Email: [call0006@student.monash.edu](mailto:call0006@student.monash.edu)

ORCID iD: 0009-0000-1256-7360

PubMED

| # | Query |
| --- | --- |
| #1 | fibroid or uterine fibroid or leiomyoma or uterine leiomyoma or endometriosis or PMS or pre-menstrual syndrome |
| #2 | progestin or progesterone or progesterone therapy or progesterone treatment |
| #3 | pain improvement or pain reduction or pain relief or treatment of pain or management of pain or adverse effects |
| #4 | "2000/01/01"[Date - Publication] : "2024/04/07"[Date - Publication] |
| #5 | 1 AND 2 AND 3 AND 4 |

CENTRAL

| # | Query |
| --- | --- |
| #1 | MeSH descriptor: [Progestins] explode all trees |
| #2 | MeSH descriptor: [Progesterone] explode all trees |
| #3 | progestin* or progestogen* or progesterone* |
| #4 | progestogen‐releasing intrauterine systems OR LNG OR LNG-IUS OR intrauterine system or mirena |
| #5 | #1 OR #2 OR #3 OR #4 |
| #6 | MeSH descriptor: [Premenstrual Syndrome] explode all trees |
| #8 | PMS or premenstrual syndrome or pre-menstrual syndrome or premenstrual tension or late luteal phase dysphoric disorder or LLPDD or premenstrual dysphoria or PMD or PMDD |
| #9 | MeSH descriptor: [Endometriosis] explode all trees |
| #10 | endometriosis |
| #11 | MeSH descriptor: [Myoma] explode all trees |
| #13 | myoma* or leiomyoma* or fibroid* |
| #14 | #6 OR #7 OR #8 OR #9 OR #10 OR #11 OR #12 OR #13 |
| #15 | pain relief OR pain reduction or adverse effects or symptom relief or symptom improvement |
| #16 | MeSH descriptor: [Pain Management] explode all trees |
| #17 | #15 OR #16 |
| #18 | MeSH descriptor: [Pregnanes] explode all trees |
| #19 | MeSH descriptor: [Norpregnanes] explode all trees |
| #20 | MeSH descriptor: [Androstanes] explode all trees |
| #21 | MeSH descriptor: [Estranes] explode all trees |
| #22 | #18 OR #19 OR #20 OR #21 |
| #23 | #22 or #5 |
| #24 | #23 AND #14 |
| #25 | #23 AND #14 AND #17 |
