# Supplementary material for: The efficacy of progestins in managing pain associated with endometriosis, fibroids and pre-menstrual syndrome: a systematic review
Source: Arch Gynecol Obstet. 2025 Mar 11;311(6):1511–33. doi: 10.1007/s00404-025-07957-0 (PMC12055938; doi:10.1007/s00404-025-07957-0)
Supplement: Supplementary file 3 — Supplementary file3 (DOCX 19 KB) [file 404_2025_7957_MOESM3_ESM.docx]

**Online Resource 3: Excluded Studies**

**The Efficacy of Progestins in Managing Pain Associated with Endometriosis, Fibroids and Pre-Menstrual Syndrome: A Systematic Review**

**Authors:** Connor Luke Allen^1, 2^. Saikat Banerjee^3^. Mahantesh Karoshi^4^. Peter Humaidan^5, 6^ . Farshad Tahmasebi^4^.

^1^Department of Medicine, Nursing and Health Sciences, Monash University, Melbourne, Australia

^2^Western Health, Melbourne, Australia

^3^CEES-u: Cambridge University Hospitals, Cambridge, United Kingdom

^4^Royal Free London NHS Foundation Trust, London, United Kingdom

^5^The Fertility Clinic, Skive Regional Hospital, Skive, Denmark

^6^Department of Clinical Medicine, Aarhus University, Denmark

Corresponding author:

Dr Connor Luke Allen

Email: [call0006@student.monash.edu](mailto:call0006@student.monash.edu)

ORCID iD: 0009-0000-1256-7360

| Author (Year) | Title | Reason for exclusion |
| --- | --- | --- |
| Telimaa (1987) | Placebo-controlled comparison of danazol and high-dose medroxyprogesterone acetate in the treatment of endometriosis | Published before 2000 |
| Sampson (1988) | Premenstrual syndrome. A double-blind cross-over study of treatment with dydrogesterone and placebo | Published before 2000 |
| Freeman (1995) | A double-blind trial of oral progesterone, alprazolam, and placebo in treatment of severe premenstrual syndrome | Published before 2000 |
| Wenzl (1993) | GnRH analogs versus gestagens in therapy of endometriosis | Published before 2000 |
| West (1990) | Inhibition of ovulation with oral progestins--effectiveness in premenstrual syndrome | Published before 2000 |
| Dennerstein (1985) | Progesterone and the premenstrual syndrome: a double blind crossover trial | Published before 2000 |
| Hammerback (1989) | The premenstrual syndrome: a study of its diagnosis and pathogenesis | Published before 2000 |
| Magill (1995) | Investigation of the efficacy of progesterone pessaries in the relief of symptoms of premenstrual syndrome. progesterone Study Group | Published before 2000 |
| Vercellini (1996) | Depot medroxyprogesterone acetate versus an oral contraceptive combined with very-low-dose danazol for long-term treatment of pelvic pain associated with endometriosis | Published before 2000 |
| Freeman (1990) | Ineffectiveness of progesterone suppository treatment for premenstrual syndrome | Published before 2000 |
| Inki (2002) | Comparison of ovarian cyst formation in women using the levonorgestrel-releasing intrauterine system vs. hysterectomy | Doesn’t specify pain reduction as an outcome |
| Cosson (2002) | Dienogest is as effective as triptorelin in the treatment of endometriosis after laparoscopic surgery: results of a prospective, multicenter, randomized study | Doesn’t specify pain reduction as an outcome |
| Iwami (2019) | Dienogest is effective for a progestin-primed ovarian stimulation protocol for in vitro fertilization while continuing the treatment of endometriosis | Doesn’t specify pain reduction as an outcome |
| Liu (2005) | The effects of progestins on bone density and bone metabolism in postmenopausal women: a randomized controlled trial | Doesn’t specify pain reduction as an outcome |
| Vahid-Dastjerdi (2023) | Comparison of the effectiveness of Dienogest with medroxyprogesterone acetate in the treatment of pelvic pain and recurrence of endometriosis after laparoscopic surgery | All patients received GnRH-a prior |
| Schultze-Mosgau (2016) | Pharmacokinetics, pharmacodynamics, safety and tolerability of an intravaginal ring releasing anastrozole and levonorgestrel in healthy premenopausal women: a Phase 1 randomized controlled trial | Not progesterone only therapy |
| Harada (2017) | Ethinylestradiol 20 μg/drospirenone 3 mg in a flexible extended regimen for the management of endometriosis-associated pelvic pain: a randomized controlled trial | Not progesterone only therapy |
| Wang (2009) | Effects and safety of gonadotrophin-releasing hormone agonist combined with estradiol patch and oral medroxyprogesterone acetate on endometriosis | Not progesterone only therapy |
| Vercellini (2002) | Cyproterone acetate versus a continuous monophasic oral contraceptive in the treatment of recurrent pelvic pain after conservative surgery for symptomatic endometriosis | Not progesterone only therapy |
