# Supplementary material for: The efficacy of progestins in managing pain associated with endometriosis, fibroids and pre-menstrual syndrome: a systematic review
Source: Arch Gynecol Obstet. 2025 Mar 11;311(6):1511–33. doi: 10.1007/s00404-025-07957-0 (PMC12055938; doi:10.1007/s00404-025-07957-0)

**Online Resource 4: Risk of Bias Assessments (Cochrane RoB 2)**

**The Efficacy of Progestins in Managing Pain Associated with Endometriosis, Fibroids and Pre-Menstrual Syndrome: A Systematic Review**

**Authors:** Connor Luke Allen^1, 2^. Saikat Banerjee^3^. Mahantesh Karoshi^4^. Peter Humaidan^5, 6^ . Farshad Tahmasebi^4^.

^1^Department of Medicine, Nursing and Health Sciences, Monash University, Melbourne, Australia

^2^Western Health, Melbourne, Australia

^3^CEES-u: Cambridge University Hospitals, Cambridge, United Kingdom

^4^Royal Free London NHS Foundation Trust, London, United Kingdom

^5^The Fertility Clinic, Skive Regional Hospital, Skive, Denmark

^6^Department of Clinical Medicine, Aarhus University, Denmark

Corresponding author:

Dr Connor Luke Allen

Email: [call0006@student.monash.edu](mailto:call0006@student.monash.edu)

ORCID iD: 0009-0000-1256-7360


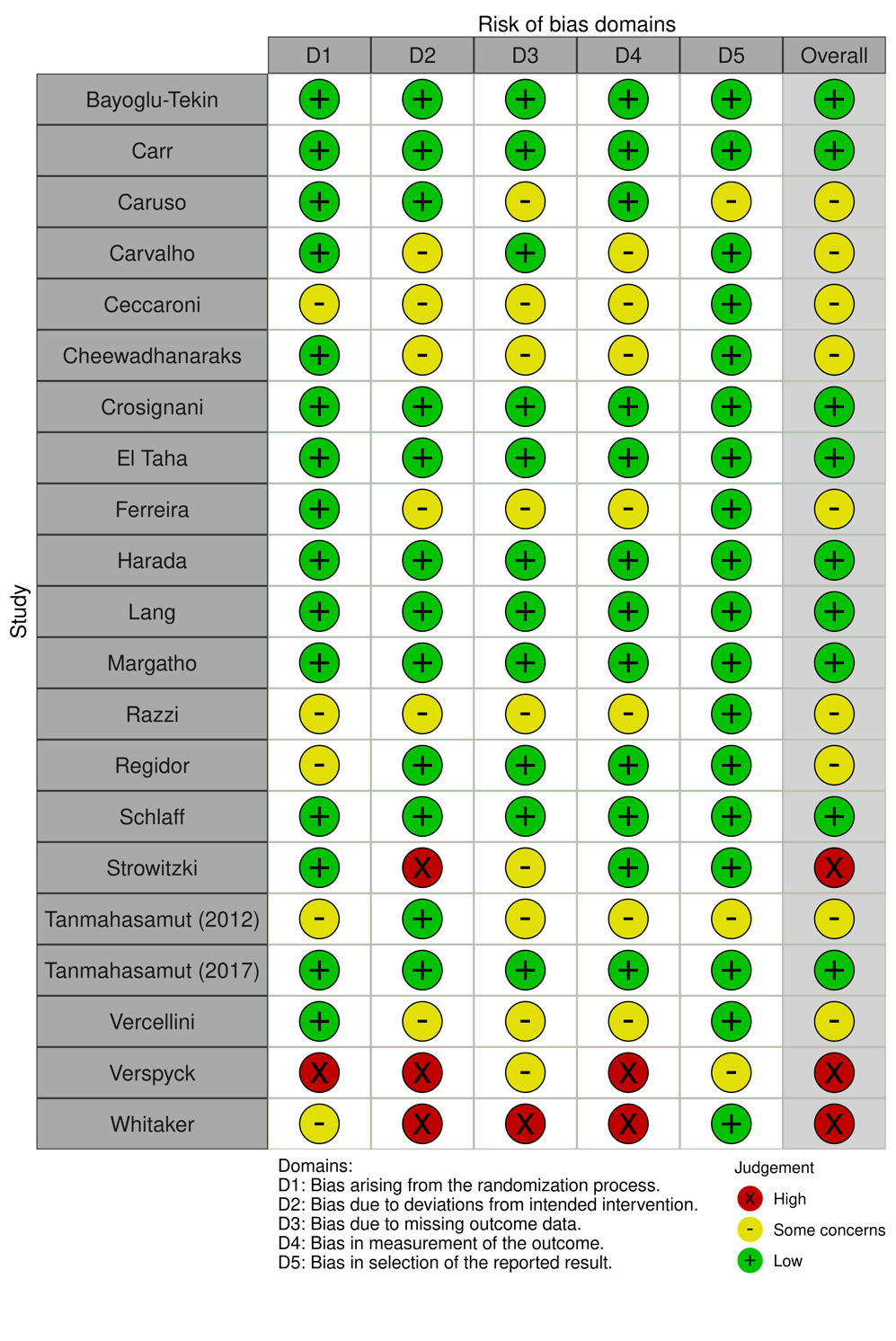


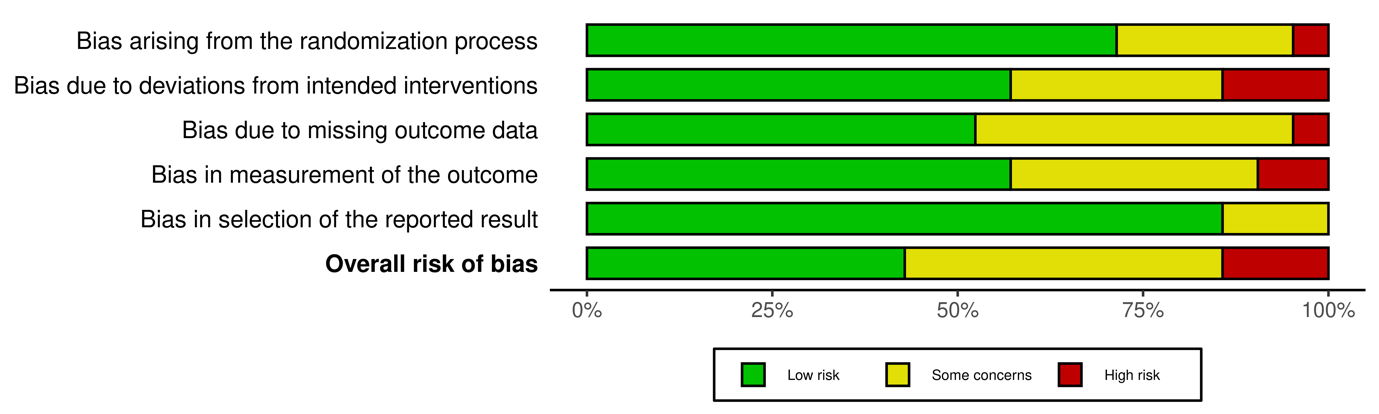

Supplement: Supplementary file 4 — Supplementary file4 (DOCX 491 KB) [file 404_2025_7957_MOESM4_ESM.docx]
